# Estimating Marginal Healthcare Costs Using Genetic Variants as Instrumental Variables: Mendelian Randomization in Economic Evaluation

**DOI:** 10.1007/s40273-016-0432-x

**Published:** 2016-08-02

**Authors:** Padraig Dixon, George Davey Smith, Stephanie von Hinke, Neil M. Davies, William Hollingworth

**Affiliations:** 1 School of Social and Community Medicine, University of Bristol, Canynge Hall, 39 Whatley Road, Bristol, BS8 2PS UK; 2 MRC Integrative Epidemiology Unit at the University of Bristol, Oakfield House, Oakfield Grove, Bristol, BS8 2BN UK; 3 School of Economics, Finance and Management, University of Bristol, 8 Woodland Road, Bristol, BS8 1TN UK

## Abstract

Accurate measurement of the marginal healthcare costs associated with different diseases and health conditions is important, especially for increasingly prevalent conditions such as obesity. However, existing observational study designs cannot identify the causal impact of disease on healthcare costs. This paper explores the possibilities for causal inference offered by Mendelian randomization, a form of instrumental variable analysis that uses genetic variation as a proxy for modifiable risk exposures, to estimate the effect of health conditions on cost. Well-conducted genome-wide association studies provide robust evidence of the associations of genetic variants with health conditions or disease risk factors. The subsequent causal effects of these health conditions on cost can be estimated using genetic variants as instruments for the health conditions. This is because the approximately random allocation of genotypes at conception means that many genetic variants are orthogonal to observable and unobservable confounders. Datasets with linked genotypic and resource use information obtained from electronic medical records or from routinely collected administrative data are now becoming available and will facilitate this form of analysis. We describe some of the methodological issues that arise in this type of analysis, which we illustrate by considering how Mendelian randomization could be used to estimate the causal impact of obesity, a complex trait, on healthcare costs. We describe some of the data sources that could be used for this type of analysis. We conclude by considering the challenges and opportunities offered by Mendelian randomization for economic evaluation.

## Key Points for Decision Makers

**Table:** 

The causal effects of health conditions on cost can be estimated using genetic variants as instruments for health conditions.
This form of analysis—Mendelian randomization—can identify causal effects because genetic variants that influence health status may be unrelated to known and unknown confounders.
Datasets with linked genotypic and resource use information are now becoming available and will facilitate this form of analysis.

## Introduction

Accurate estimates of the marginal medical healthcare costs that are incurred as a consequence of specific health conditions are important. Estimates of cost are fundamental to the economic evaluation of healthcare technologies, whether undertaken alongside randomized controlled trials (RCTs) [1] or as an element of decision-analytic modelling [2, 3]. Health system sustainability depends on an understanding of changes in population health and associated healthcare costs [4–6].

Neither observational studies nor RCTs offer a wholly satisfactory means of estimating the impact of different health conditions on cost. Observational studies can estimate the correlations between healthcare costs and health conditions but generally cannot identify causal relationships [7]. It is particularly difficult to infer causal effects of specific conditions on healthcare costs because of prevalent comorbidities and common causes of health outcomes and healthcare costs such as socioeconomic status (confounding), complicated natural histories (reverse causality) and self-reported health status (measurement error).

Many of these problems cannot be resolved in RCTs. Trials are rarely powered to detect differences in cost-related outcomes [8]. It may be neither feasible nor ethical to expose patients to the risks of an intervention solely to collect information on cost associated with different health conditions [9]. Patients recruited to RCTs may not be representative of the populations concerned. Cost data collected in RCTs may have limited generalizability, may not relate to the costs that would arise in routine practice, may be related to intermediate rather than final outcomes and may not be collected for the full period over which a health condition affects cost [10].

We describe recent developments in genetic epidemiology that offer a new way of estimating the causal impact of health conditions on healthcare cost. The methodology of Mendelian randomization, which uses genetic variants as instrumental variables, offers a means of addressing the limitations of existing study designs. In particular, the ethical and feasibility issues that would prohibit the conduct of an RCT are avoided, but some of the advantages of interventional studies in relation to causal inference are retained.

We illustrate how robust estimates of causal effects of health conditions on costs could contribute to economic evaluation, and health economics more generally, by considering the relationship between obesity and cost as a motivating example throughout the paper. Obesity is an increasingly prevalent condition [11] that is associated with a range of adverse health [11] and economic [12] outcomes.

Improved estimates of the causal relation between health conditions (such as obesity) and healthcare cost could offer important new evidence to at least three important areas of health economics. The first area is decision-analytic modelling. Decision-analytic models, which synthesise information from a variety of sources, including observational studies and RCTs, are increasingly recommended as the most appropriate vehicle for cost-effectiveness analysis [13]. For example, simulation of the lifetime consequences of obesity requires information on the cost consequences of different health states that are defined by body mass index (BMI). The conclusions of these studies are likely to be more secure if they are informed by robust causal evidence.

The second area is health system management. For example, in the absence of accurate information on the cost consequences of obesity, how should healthcare funders react to information that indicates the prevalence of obesity is expected to continue to increase?

The third area relates to targets for intervention. For example, if an apparent association of BMI with cost is actually confounded by an association of obesity with mental health status, then an intervention targeted solely at reducing adiposity is likely to be neither effective nor cost effective. Improved knowledge of causal relationships will help avoid wasteful research effort and facilitate the setting of research priorities [14].

The objective of this paper is to provide an overview of the potential role of Mendelian randomization in estimating the causal effect of health conditions on healthcare cost. We begin by briefly describing the need for improved methods for causal inference when analysing observational data, then set out the genetic basis for Mendelian randomization and its relationship to instrumental variables analysis. We describe the key conditions that must be met for this form of instrumental variable analysis to produce valid causal estimates of effect, with particular regard to the specific issues and biological contexts that arise when analysing genetic variants as instrumental variables.

To make this concrete, we describe the methodological issues that would arise when attempting to use Mendelian randomization to estimate the causal effects of obesity on healthcare cost. We use this example throughout the paper to link together the methodological issues. We then consider the data sources that could facilitate this type of analysis, which would represent a novel use of the large linked genomic cohort datasets that are now being developed and made available to researchers.

We conclude by summarising some the challenges and opportunities offered by Mendelian randomization for the causal analysis of cost.

## Genetic Variants and Instrumental Variables Analysis

### Rationale

**Fig. 1:**
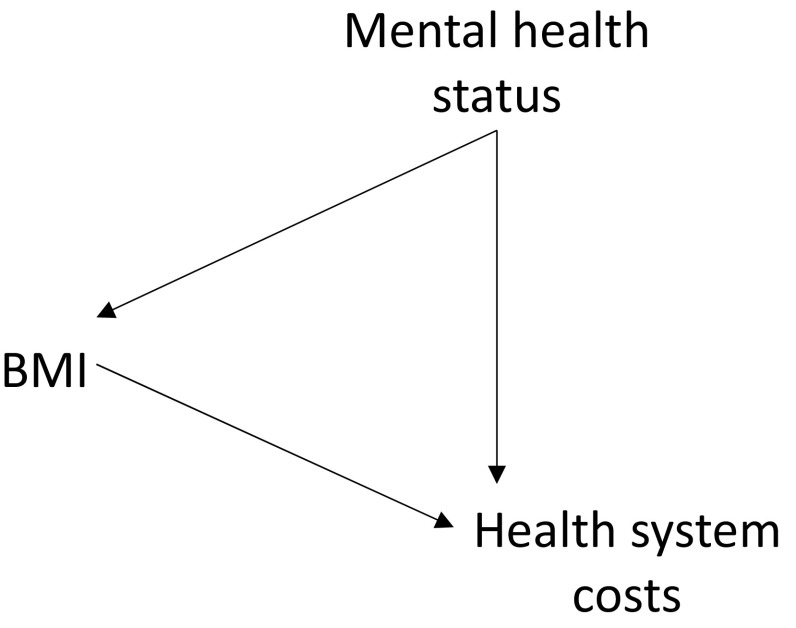
A relationship between an exposure (body mass index [BMI]) and an outcome (healthcare costs) that is subject to confounding (e.g. by mental health status)

The rationale for undertaking causal analysis of the form described below is that the relationship of some exposure (such as BMI) to an outcome (such as healthcare costs) is known or suspected to be confounded. Figure 1 illustrates this situation using a directed acyclic graph [15].

Figure 1 shows that BMI is related to healthcare costs, but a third variable (or variables) influences both BMI and costs. For example, mental health may be unobserved, but if individuals with worse mental health are more likely to have higher BMI and (independently) have higher healthcare costs, the relationship between BMI and costs is confounded. However, in general, these confounding variables may be unknown, known but measured with error, or known but not quantifiable. A simple linear regression of outcome on exposure would not identify the causal influence of exposure in the presence of confounding.

In the next section, we describe how instrumental variable analysis using genetic variants can offer a means of identifying the causal effect of an exposure (such as obesity or BMI) on an outcome (such as healthcare costs). We begin by providing some biological context.

### Genetics and the Basis for Mendelian Randomization

The human genome is made up of 23 pairs of chromosomes located in the nucleus of almost every cell in the human body. Chromosomes are made up of molecules of deoxyribonucleic acid (DNA), which is constituted (in part) by nucleotides, themselves comprising nucleobases: cytosine (C), guanine (G), adenine (A) and thymine (T) [16].

The region of the chromosome at which a specific genetic variant in a DNA sequence is located is called its locus [17]. Each locus in the human genome contains two alleles; an allele is the particular form of a gene. Single nucleotide polymorphisms (SNPs) refer to an individual locus that varies across people in a population. SNPs may occur during cell division (meiosis). Other forms of variation are possible, but most commonly researched genetic variants are SNPs [18].

Individual SNPs can affect different observable traits such as disease status or health condition. This introduces the distinction between heredity (the genotype) and the consequences of that heredity (the phenotype). The phenotype can be thought of as an ‘outward’ characteristic or trait that can be observed and/or measured, while the genotype is the underlying genetic structure associated with a specific phenotype [16].

Mendelian randomization is founded on Mendel’s first and second laws. The first law—the principle of segregation—states that, during the formation of sex cells (gametes), there is random segregation of alleles from parent to child. The second law is the independent assortment of genetic variation at conception. This ‘allocation’ of genetic variation at the time of conception is approximately random, conditional on parental genotype. Genetic variants that do not affect an outcome of interest other than through the (phenotypic) risk exposure/health condition with which they are known to be associated can serve as instruments to allow researchers to infer the causal effects of health conditions on outcomes of interest, such as healthcare cost [19]. Individual genetic variants may therefore be valid instrumental variables.

In the language of econometrics, the effects of the endogenous variable (health condition) can be identified by the exogenous variation induced by the genetic variants. The genetic variants are assumed to be orthogonal to a regression error term because of the approximately random allocation (no confounding or endogeneity) at the time of conception (no reverse causality) and the absence of measurement error in the (more precisely measured) genetic variant instruments. Genome-wide association studies (GWAS) increasingly provide robust evidence concerning the association of individual genetic variants and phenotypes. Thousands of such associations have now been identified [20], and research continues [21].

An analogy may be drawn with an RCT. In Mendelian randomization, allocation to ‘treatment’ is indicated by the genotype, which is known to be associated with the health condition of interest. Differences in outcomes in people with different genotypes may then be investigated in a manner equivalent to an intention-to-treat (ITT) analysis in an RCT, in which participants are analysed irrespective of their compliance with the intervention [22].

An important difference between Mendelian randomization and RCT analysis is that the genotype of interest represents a lifelong difference in the health condition or risk factor concerned, rather than the effects of a short-term administered intervention. Mendelian randomization can potentially estimate the effects of a health condition when intervention studies would be unethical or impractical, e.g. the assignation of individuals to alcohol dependence or to obesity.

### Genetic Variants and the Assumptions of Instrumental Variables Analysis

Many reviews of Mendelian randomization methodologies are available [14, 18, 22–25]. This section briefly reviews three fundamental assumptions of instrument variable (IV) analysis. These are the same whether the instrument is a genetic variant or any other (non-genetic) variable.

The three core instrumental variable assumptions are (1) relevance (the instrument must be associated with the exposure), (2) independence (the instrument is not associated with confounders) and (3) exclusion (the instrument does not directly affect the outcome).^1^ Further ‘point identifying’ assumptions, such as monotonicity and ‘no effect modification’, may also be required and are discussed elsewhere [18, 24, 26–28].

**Fig. 2:**
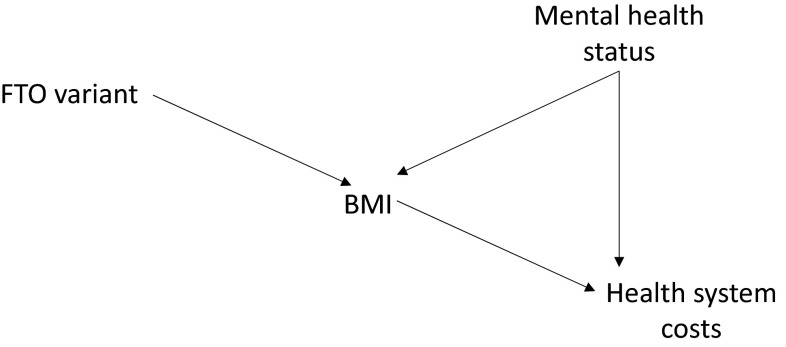
A relationship between an exposure (body mass index [BMI]) and an outcome (costs) that is subject to confounding (by mental health status) but for which a valid instrumental variable (the *FTO* variant) exists

Figure 2 illustrates a situation where the three IV assumptions described above are fulfilled by an instrumental variable. A Mendelian randomization analysis could use variants of the *FTO* gene [29, 30], which are known to be associated with obesity, as an instrument to estimate the causal effects of BMI on costs.

In Fig. 2, the instrumental variable (the *FTO* variant) is related to the exposure (BMI), indicated by the arrow pointing from FTO to BMI, and thus the first IV assumption holds. The confounding variable does not influence the instrument (or vice versa) since there are no arrows between mental health status and the FTO variant. Thus, the second assumption is satisfied. Finally, the only arrow leading from the instrument is to BMI—the instrument influences costs only via this path and does not otherwise affect the outcome, as required by the third IV assumption.

#### The Relevance Assumption

Genetic variants must have a robust association with the exposure of interest to be valid instrumental variables. This is known as the relevance assumption [31]. Instrumental variable estimates may be biased when an instrument explains only a small part of the variation in the exposure [32, 33]. Using genetic variants that have been robustly associated with the exposure in large replicated genome-wide studies can avoid biases that can arise when choosing genetic variants whose association with the exposure has been demonstrated only in a single dataset [34]. Bias can also arise when a measured exposure is an imperfect proxy for an underlying exposure. Taylor et al. [34] discuss this possibility with an example concerning self-reported cigarette consumption as an imperfect proxy for actual cigarette consumption.

The relevance assumption can be tested by estimating the association of the variants and the exposure [28]. These tests could also account for gene–environment interactions. For example, the relationship between phenotypes and variants that influence the consumption of food may be concealed in contexts where little calorific food is available [14].

#### Independence Assumption

The independence assumption refers to the independence of the instrument from all confounders. Intuitively, this can be understood as the variants being ‘as good as’ randomly assigned to different individuals.

Population structure can induce associations between genetic variants and outcomes (e.g. cost) that are not due to the effect of the exposure of interest (e.g. BMI). This can occur because of population stratification, by which population subgroups differ in their relationship between the exposure and outcome. For example, allele frequencies of *FTO* are known to vary by ethnic group. If these ethnic groups also have systematically different healthcare costs for reasons other than obesity, the independence assumption is violated. This can be accounted for by stratification of the population according to the subgroup, limiting analysis to groups with similar ancestral backgrounds or adjusting for ancestry-informative principal components [14].

Assortative mating, the preferential mating of like genotypes (driven by mating of like phenotypes), will also tend to isolate alleles in certain population subgroups [16]. Assortative mating can violate the independence assumption and introduce bias into Mendelian randomization, since genetic variants may be confounded by associations with the behavioural or social factors that characterise these population subgroups.

#### Exclusion Restriction Assumption

The exclusion restriction is so called because a valid instrument can be thought of as ‘excluded from’ or ‘exogenous to’ the causal relationship of interest by virtue of having no direct effect on the outcome or by being unrelated to any other determinant of the outcome [26].

The exclusion restriction can be violated in the presence of canalization [35], which refers to compensation for the effects of the variant(s) under investigation. This can cause estimated effect sizes to be attenuated. Canalization could reflect changes during gestation or environmental forces that buffer the consequences to the individual of the health condition under study.

The interpretation of analysis in this context is similar to ITT analysis in an RCT, with canalization playing a role comparable to non-adherence to an intervention. Gene-by-environment interactions could provide evidence that particular exposures are affected by canalization. This is because development will not usually occur in the presence of a modifiable risk factor; hence, no compensation could have occurred [36]. However, the availability of datasets to conduct well-powered studies of these interactions is limited [37].

**Fig. 3:**
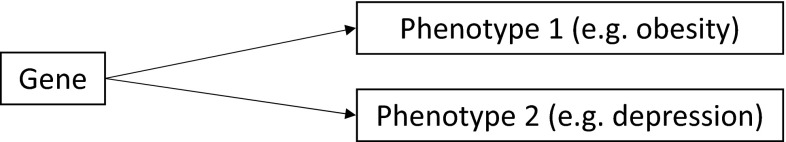
Pleiotropy—a gene that affects more than one phenotype

Pleiotropy refers to the phenomenon in which a single locus directly or indirectly affects more than one phenotypic trait [14, 22, 38]. Figure 3 provides a simple representation [39].

Pleiotropy may violate the exclusion assumption. For example, assume a hypothetical gene separately influences both obesity and depression. A Mendelian randomization analysis using this gene to assess the causal effects of obesity could be confounded inadvertently by depression if both traits affect the outcome of interest.

**Fig. 4:**
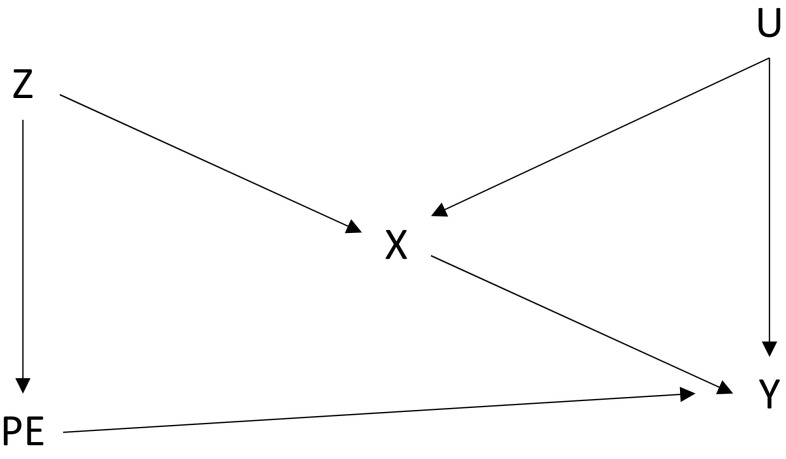
A confounded pleiotropic variant. *PE* pleiotropic effect, *X* health condition, *U* confounding variable(s), *Y* healthcare cost, *Z* instrumental variable

This type of scenario is summarised in Fig. 4 (based on Lawlor et al. [40]), in which U is a confounding variable and the pleiotropic effect (PE) creates a pathway for the variant (Z) to influence the outcome (Y) other than through the exposure (X).

Note that, even if a variant is pleiotropic, it need not violate the exclusion restriction, provided that the other trait does not affect the outcome (i.e. if there is no line from PE to Y in Fig. 4).

Clear understanding of genetic function is one source of protection against pleiotropic confounding [37]. Evidence from multiple IV models that use different combinations of variants to predict the same causal effect is another [19, 24, 41]. For example, if many variants (not in linkage disequilibrium) imply the same causal effect, then pleiotropy is unlikely to explain the results. This is because the same causal effect across different variants could have been obtained only if the pleiotropy operated in such a way as to ‘cancel out’ under- and overestimates of effect [37].

**Fig. 5:**
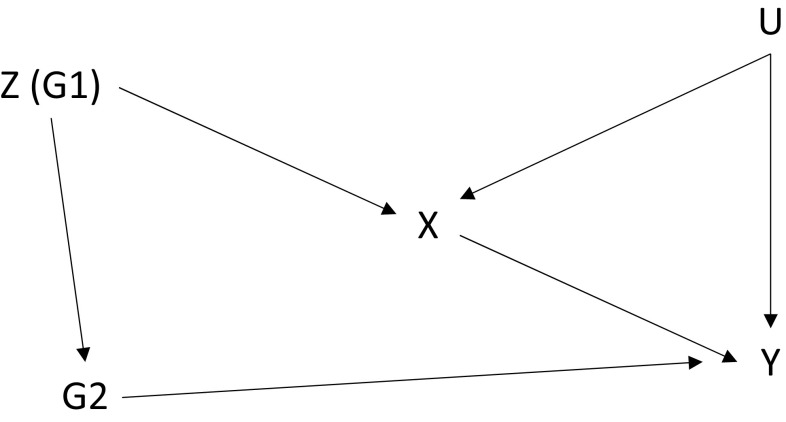
A variant (G1) in linkage disequilibrium with another variant (G2) that also affects the outcome. *X* health condition, *U* confounding variable(s), *Y* healthcare cost, *Z* instrumental variable

Co-inheritance of traits, against Mendel’s second law, may also violate the exclusion restriction [18, 35]. One example is linkage disequilibrium, which occurs when genetic variants tend to be inherited together, so that variants other than those under study contribute to the trait. This is illustrated in Fig. 5, which can be compared with the illustration of pleiotropy in Fig. 4. In Fig. 4, the variant is connected with a *trait* that affects the outcome of interest; in Fig. 5, the variant (denoted G1) is connected with another *variant* (denoted G2) that itself affects the outcome [40]. For example, if *FTO* tends to be co-inherited with a variant that predicts a mental health condition, which independently affects healthcare costs, the exclusion restriction is violated.

The testing of suspected associations and recourse to evidence on genetic function and known linkages can offer protection against violations of the exclusion restriction caused by linkage disequilibrium. For example, ‘maps’ of the human genome can provide information on linkage disequilibrium [42]. Linkage disequilibrium need not be problematic, provided that the second variant (G2 in Fig. 5) does not affect the outcome (no line from G2 to Y).

The exclusion restriction can be examined indirectly by establishing whether the proposed genetic variants are associated with potential confounding factors, or alternative mediating variables, though one cannot directly test whether the exclusion restriction is valid. If either or both of these associations are observed, then the exclusion restriction is unlikely to hold. However, inference can still be undertaken even if some instruments are invalid, as discussed below in Sect 2.5. Pischke and Schwandt [43] noted that regressing suspected confounders on included variables can be more informative than regressing the outcome of interest on suspected confounders if confounders are poorly measured.

Genetic variants are generally not related to confounders that affect observational studies. For example, Davey Smith et al. [44] found that a variety of behavioural, socioeconomic and physiological phenotypic variables are strongly correlated, but genetic variants were not correlated either with each other or with the phenotypic variables beyond what would be expected by chance.

However, dynastic effects, in which a genetic trait of a child is affected by a parental exposure caused by the parental genotype, can confound variants [45]. For example, if a variant carried by a parent causes increased adiposity, and this causes a parent to avoid exercise with their children, then both the variant for adiposity and a behavioural tendency to avoid exercise would be passed on to the offspring. This could confound the effect of the variant. Between-sibling (or within-family) Mendelian randomization would offer a solution to this.

### Estimating Strategies for Undertaking Mendelian Randomization Analysis

The Wald estimator, or the ratio method, involves calculating the ratio of estimated coefficients obtained from a regression of the outcome on the instrument to the coefficient obtained from a regression of the exposure on the instrument. The intuition here is that a unit change in the outcome for a unit change in the exposure is given by the ratio of an ‘effect’ of the instrument on the outcome to an ‘effect’ of the instrument on the exposure. The familiar two-stage least squares (2SLS) estimator will give the same estimated causal effect as the ratio method when using a single instrument [28].

Mendelian randomization can be conducted using likelihood-based estimators, with Bayesian methods, and semi-parametric methods such as the generalized method of moments (GMM) and structural mean models (SMM). Burgess et al. [28] argued that “there is no single universal ‘best’ IV estimation method. Instead, the use of different IV methods provides sensitivity analyses to assess whether the estimate given by a particular choice of method is credible.”

The precision of IV estimates will generally be less than that of corresponding observational estimators, since the size of IV standard errors is inversely related to the strength of the association between instrument and exposure. The greater consistency of an IV estimator in the presence of confounding is associated with wider confidence intervals around estimated effect size.

### Inference with Invalid Instruments

Recent methodological developments offer the possibility of obtaining unbiased estimates of the causal effect of exposure, even when some or all of the proposed variants are invalid.

For example, Bowden et al. [46] proposed a form of Mendelian randomization analysis that can provide consistent estimates of the treatment effect even if the variants have pleiotropic effects. Do et al. [47] considered the detection of causal influences in the presence of pleiotropy and proposed a two-stage linear regression approach for summarised data that gives separate estimates for different risk factors. This type of approach was developed by Burgess et al. [48, 49], who described a multivariable approach to Mendelian randomization that allows variants associated with more than one risk factor to be used in simultaneous estimation of the causal effect of each risk individual factor. Kang et al. [50] demonstrated that causal effects can be identified and estimated using a generalization of GMM estimators, even where there is no knowledge about which specific instruments may be invalid, provided that less than half of the instruments used in an analysis are actually invalid.

### Phenotypic Data

Phenotypic data could be drawn from medical records or other sources such as routinely collected data [51–53]. Phenotypic data can be used as a means of overcoming some of the challenges of Mendelian randomization, as an additional source of evidence on which Mendelian randomization analysis of healthcare costs might be performed and as an informative body of evidence in its own right [54]. Phenome-wide association studies (PWAS) indicate diseases associated with genetic variants, whereas GWAS identify variants associated with disease [53, 55].

Evidence from PWAS can identify associations not already known from GWAS [56] but can also validate associations [57] and provide additional evidence on pleiotropy [57, 58]. The challenges of medical records as a data source include inconsistencies in coding, coverage and the diversity of sources and systems [59].

### Instrument Variable Analysis in Mendelian Randomization

IV analysis in Mendelian randomization needs to reflect underlying biological relationships and understanding of gene function. Results need to be interpreted in a manner that reflects the functional biological context and the broader population from which data are drawn. Glymour et al. [60] encouraged ‘aggressive’ evaluation of research design, encompassing testing of the validity assumptions, evaluation of biological context and consideration of the evidence available. Burgess et al. [61] suggested using the Bradford Hill [62] criteria^2^ as a basis for judging the plausibility of the IV assumptions in Mendelian randomization analysis.

## Practical and Methodological Considerations in Causal Analysis of a Complex Trait

In this section, we illustrate some of the practical and methodological issues that might affect a Mendelian randomization analysis of the effects of obesity on healthcare costs.

The prevalence of obesity has increased in recent decades [11, 63]. It is associated with high healthcare costs [7], is often comorbid [64], and is known to have a heritable component [67]. Furthermore, BMI measurement error is pervasive [65] and may be substantial [66]. Literature using Mendelian randomization to examine the relationship between BMI and health/non-health outcomes is extensive [24, 68–73].

If obesity is a notable trait on these grounds, it is also a challenging one. Obesity is a complex trait—many genetic variants affect BMI [71]. The use of genetic variants might violate the IV assumptions in a number of ways. We discuss these in more detail below in Sect 3.1–3.4, outlining the main methodological issues.

### Weak Instruments

The standard errors of IV estimators are related to the strength of association between instrument and exposure. Multiple instruments—such as the many genetic variants known to be associated with obesity [74]—can improve statistical power. The intuition for this is that if multiple instruments are available and orthogonal to regression errors, then a linear combination will also be orthogonal [75].

However, multiple weak instruments will bias the IV estimates toward the observational estimate [18, 76]. Burgess and Thompson [18] suggested this bias can be alleviated by using parsimonious models of genetic association, such as allele scores. Allele scores are weighted or unweighted variables that combine into a single variable information from multiple genetic variants, and this (use of external information) can increase the power of IV analysis.

All the variants in the score must be valid instruments for an allele score to meet the IV assumptions; even minor violations of the exclusion restriction can introduce bias into approaches using single allele scores. Davies et al. [32] suggested the use of the continuously updating estimator as a means of addressing weak instruments. This estimator can be used in circumstances with multiple risk factors and many variants in which it would be difficult to create different allele scores for each risk factor.

### Multiple Samples

The data on which estimation is performed need not come from a single sample [22]. Data on the exposure/outcome association (such as obesity and healthcare costs) and variant/exposure relationship (a genetic variant and obesity) could, in principle, be estimated on different samples.

All of the assumptions described concerning validity of analysis continue to apply, and particular care needs to be taken to ensure that the populations in each study are comparable [28]. The absence of individual-level data will restrict the types of analyses that may be conducted and the ability to test the IV assumptions may be diminished when multiple samples are used [77, 78].

### Non-Linearity

Some relationships of interest to health economists, such as between BMI and healthcare costs, are likely to be non-linear [7]. Where exposure–outcome relationships are not approximately linear, then instrumental variable estimates using a linear model may not reflect causal effects for large changes in the exposure [79]. If the exposure–outcome relationship is both non-linear and non-monotone, even small changes in values of the exposure will be difficult to interpret [18].^3^

If the shape of the exposure–outcome relationship is of interest, and its association between exposure and genetic association is the same at different levels of the exposure, then stratification within different quantiles of the exposure can be performed to examine the local impacts of the exposure on the outcome, although stratification should not be directly on the exposure itself to avoid inducing an association between the IV and confounders [28, 79]. Silverwood et al. [80] described a related method for estimating local average treatment effects for discretized values of the exposure.

### Healthcare Costs as an Outcome in Mendelian Randomization

Linking healthcare costs to a specific health condition can be complicated. For example, Lehnert et al. [81] noted that the physical burden of adiposity itself is not the major source of economic burden on the individual or on health systems. Instead, this burden is mostly attributable to medical conditions that originate from endocrinal and metabolic changes, such as type 2 diabetes mellitus and cardiovascular disease.

This gives rise to a conceptual question: should the causal analysis of the cost consequences of obesity focus on total healthcare costs or on ‘obesity-related’ costs only? Casting the net widely to encompass total costs allows for unknown and unexpected influences on cost causally related to the variant and exposures of interest to be included in the analysis. Consider an example of an individual who experiences a car accident, to which diabetic retinopathy associated with obesity contributed, and who undergoes an expensive inpatient hospital stay. A focus on ‘obesity-related’ costs that excluded consideration of this type of emergency admission could overlook these costs, even though they are caused by obesity in the scenario described. Both a total cost approach and an obesity-specific approach could be undertaken if information on overall resource use and resource use by diagnostic code is available.

### Data Sources

An ideal data source for the type of analysis proposed in this paper would contain extensive genotypic information on as large a group of individuals as possible, linked to longitudinal medical records and/or routinely collected administrative claims or reimbursement data. We focus on the UK Biobank project as an example dataset.

The UK Biobank is a prospective study of approximately 500,000 participants aged between 40 and 69 years at recruitment between 2006 and 2010 [82]. Detailed phenotypic and genotypic data are being collected from diverse sources, including questionnaires, assays, imaging and genotyping [82]. As of early 2015, approximately 8500 deaths and 600,000 hospital admissions had also been recorded via routinely collected data beginning in 1997. Hospital outpatient episodes from 2003 onwards were included in 2015, and primary care data will be added in the future [82].

Part of the UK Biobank’s motivation in recruiting individuals aged at least 40 years was to ensure a sufficient number of incident outcomes during the early years of follow-up. There is likely to be a minor selection effect in observing the health outcomes of individuals who have survived to at least 40 years of age.

A more severe issue of selection relates to participation in the study itself. Participants in the UK Biobank face lower mortality risks than the general population. This gives rise to the potential for selection bias (a form of collider bias) [83], whereby the associations observed between genetic variants and cost could differ from the relationship in the general population. This is because the characteristics that give rise to selection into the study may affect exposures, and thus exposure–cost relationships. The incorporation of routine biological sample collection into population-based databases with wide coverage would help improve generalisability in other study contexts.

### Textbox 1 Examples of Other Datasets

Studies with wide population coverage include the Age, Gene/Environment Susceptibility-Reykjavik Study, which contains linked genetic, phenotypic and medical records data for a large Icelandic cohort [82]. The Estonian Biobank contains similar information on 5 % (approximately 52,000 individuals) of the Estonian adult population [83].

The UK 100,000 Genome Project plans to sequence 100,000 genomes by 2017. The project has a focus on rare diseases and on cancer [84, 85]. This will facilitate Mendelian randomization analysis on these topics, but statistical power may be limited in some cases. Linkages to routinely collected data is planned, and issues of generalisability will again need to be considered.

The eMERGE (electronic MEdical Records and GEnomics) network in the USA [84] offers links from over 55,000 participants between DNA repositories and electronic medical records. Kaiser Permanente is building a biobank of 500,000 Californian health plan members that will link medical records and genetic, behavioural and environmental data [85]. The US Department of Veterans Affairs is overseeing the Million Veteran Program, which will create a database of genetic information and medical care on 1 million volunteers [86].

## Discussion

There is scepticism that Mendelian randomization can offer anything to the study of economic outcomes [87] in spite of a number of studies that have successfully used Mendelian randomization to address ‘economic’ questions [24, 41, 50, 88]. We have outlined some of the challenges that would complicate an analysis of the causal effects of BMI/obesity on healthcare costs. This overview of challenges is not comprehensive and may vary from experiences involved in analysing other traits, but it illustrates realistic aspects of analysis that would likely be encountered.

As with RCTs, the generalizability of a Mendelian randomization analysis is not secured merely by conducting a well-designed study. For example, genetic variants tend to have modest effects on the exposures of interest, albeit that they influence lifelong exposure and not the short-term exposures often observed in RCTs, which may also produce small effect sizes [19].

RCTs and well-designed prospective cohort studies will continue to be an important source of evidence. However, there is little or no prospect of obtaining robust causal cost estimates associated with long-term exposure to many medical conditions [24]. In circumstances where the consequences of the condition of interest on cost (or some other outcome) are likely to be material, and considerations such as measurement error, reverse causality and confounding will severely affect observational analyses, then the case for Mendelian randomization analysis will be stronger.

## Conclusion

A comparison is sometimes drawn between the human genome and a book [89]—the 23 pairs of chromosomes are chapters, the texts of which are combinations of the nucleobase ‘letters’: C, G, A and T. Variations between individuals or chromosomes in single letters of text at particular parts of these chapters may have consequences for health. Mendelian randomization is the analysis of this variation using instrumental variables to make claims about aetiology and outcomes. We have outlined how Mendelian randomization could be used to understand the consequences for costs of different health conditions, focusing on obesity in particular.

Substantial progress has been made in Mendelian randomization-based analyses [23, 37]. This progress has been driven by new and large data resources, the volume of evidence emerging from GWAS, and identification and resolution of methodological challenges.

Mendelian randomization analysis is potentially a valuable technique for health economists. Contextual reasoning, large sample sizes (including multi-sample designs), a focus on SNPs with material functional consequences, evidence from a variety of sources, information on biological plausibility, and sensitivity testing could form elements of a well-designed Mendelian randomization study. The outputs of these kinds of study could support the development of more robust evidence for economic evaluations and for healthcare priority setting more generally.

## Footnotes

1 The independence and exclusion restrictions are closely related and are sometimes described as the same assumption, e.g. in Angrist and Pischke [26].

2 Briefly, the Bradford Hill criteria (as discussed by Burgess et al. [61] in relation to Mendelian randomization analysis) are that, for an exposure to have a causal impact on an outcome, then any association between the two should (1) be strong, (2) be consistent (i.e. observed in different places and at different times), (3) be specific, (4) be temporal, (5) exhibit a biological gradient, (6) be plausible, and (7) be coherent (i.e. should reflect known facts of the natural history of the disease and of biology more generally.

3 These considerations apply to IV and non-IV approaches.
